# The use of monocyte subset repartitioning by flow cytometry for diagnosis of chronic myelomonocytic leukaemia

**DOI:** 10.1038/s41408-020-00401-3

**Published:** 2021-01-07

**Authors:** Aarya Murali, Donna Cross, Peter Mollee

**Affiliations:** 1 School of Medicine, University of Queensland, Brisbane, QLD Australia; 2 Department of Haematology, Princess Alexandra Hospital, Brisbane, QLD Australia

**Keywords:** Diagnosis, Haematological cancer

Dear Editor,

We note with interest Pophali et al.’s^1^ paper examining the value of monocyte subset repartitioning by multiparametric flow cytometry (MFC) to distinguish chronic myelomonocytic leukaemia (CMML) from other causes of monocytosis. The original paper by Selimoglu-Buet et al.^2^ in 2015 noted a relative predominance of classical or MO1 monocytes (CD14+/CD16−) at the expense of MO2 (CD14low/CD16+) and MO3 (CD14−/CD16+) monocytes in patients with CMML. These authors suggested that an MO1 percentage cut-off of >94% could predict the diagnosis of CMML with high sensitivity and specificity (both >90%) whereas Pophali et al. were unable to replicate these findings, calling into question the utility of flow cytometry to distinguish the aetiology of monocytosis in a real-world setting. We wish to add data from our own experience to shed further light on this issue.

In this study, we assessed peripheral blood samples from 35 patients presenting with a monocytosis (absolute monocyte count >1 × 10^9^/L) in a tertiary referral hospital in Brisbane, Australia. The patients’ final clinical diagnosis was extracted from the medical record by two clinicians (A.M. and P.M.) blinded to the results of the flow cytometry. Ethical approval for this study was obtained from the Metro South Hospital and Health Service Human Research Ethics Committee [HREC/2020/QMS/65502]. A detailed description of the methods of flow cytometry applied in this study is available in supplementary materials.

Of the 35 patients included, 13 patients had CMML and 4 patients were diagnosed with another underlying myeloid neoplasm: one patient with myelodysplastic syndrome (MDS), one patient with myeloproliferative neoplasm-not otherwise specified (MPN-NOS), one patient with multiple myeloma and one patient with acute myeloid leukaemia with myelodysplasia related changes (AML-MRC). Table 1 summaries the individual characteristics of the 13 patients diagnosed with CMML (note: next-generation sequencing panels were not performed during the time frame of this study).

**Table 1:** Characteristics of patients with CMML (*n* = 13).

Patient ID	CMML subtype	Bone marrow cytogenetics	AMC	MO1%	MO2%	MO3%	Previous treatment for CMML	Meeting criterion for t-MDS/t-MPN
10	CMML-1	46XY, NAD	1.24	93	6	<1	No	No
11	CMML-1	46XX, NAD	8.15	96	4	<1	No	No
15	CMML-2	46XX, NAD	1.4	74	21	4	No	No
16	CMML-2	46XY, NAD	0.69	35	4	40	Yes^a^	No
17	CMML^b^	N/A	2.38	95	4	1	No	No
18	CMML^b^	N/A	2.19	95	4	1	No	No
20	CMML-2	Not done	5.05	94	6	<1	No	Yes^c^
21	CMML-1	Not done	5.64	58	35	7	No	No
23	CMML-2	46XX, NAD	36.51	98	<1	<1	Yes^d^	No
25	CMML-1^b^	N/A	3.66	95	2	2	No	No
28	CMML (subtype not specified)	46XY, NAD	4.44	97	1	2	No	No
31	CMML^e^	N/A	7.48	74	13	12	No	No
32	CMML^f^	N/A	6.48	95	2	2	No	No

^a^Monocyte flow cytometry was performed at Cycle 1 Day 7 of Azacitadine for treatment for CMML.

^b^Diagnosis of CMML was made at external institution; unable to locate report of original bone marrow aspirate and trephine.

^c^Previous breast cancer (1994) treated with lumpectomy and radiation therapy; previous diagnosis of polycythaemia rubra vera, treated with hydroxyurea.

^d^CMML previously treated with hydroxyurea—ceased after splenectomy for splenic infarction, approximately 8 months prior to monocyte flow cytometry.

^e^Bone marrow aspirate and trephine were not performed due to significant co-morbidities including dementia.

^f^Bone marrow aspirate and trephine were not performed as declined by patient.

Furthermore, six patients had a reactive monocytosis in the setting of autoimmune disease, including granulomatosis with polyangiitis (GPA) (two patients), rheumatoid arthritis (RA) (one patient), IgG4 disease (one patient), mixed connective tissue disease (MCTD) (one patient), and polyarticular gout (one patient). Lastly, 12 cases of reactive monocytosis were attributed to infective and inflammatory causes.

Among our 13 patients with CMML, 7 cases (53.8%) had an MO1 percentage >94%. Among the six patients with CMML who had an MO1 percentage ≤94%, five patients had another underlying inflammatory or autoimmune condition, including asbestosis (patient ID 10); anti-phospholipid antibody syndrome (patient ID 15); granuloma annulare (patient ID 16); urosepsis, ocular shingles and polycythaemia rubra vera (patient ID 20); and lastly, polyarteritis nodosa (patient ID 21). These patients had an MO2 fraction which ranged from 4 to 35% (Table 1). All but two patients with CMML were treatment naïve at the time that monocyte subset repartitioning was performed. Only one patient met criterion for therapy-related MDS/MPN.

In comparison, only 4 (18.2%) among the 22 cases of non-CMML were identified to have an MO1 percentage >94%—this included two patients with autoimmune disease (GPA, MCTD), one patient with MDS and one patient with recurrent infections.

There was no significant correlation between an MO1 percentage cut-off of >94% and a diagnosis of CMML (*p* = 0.057). We also examined the utility of the MO3 percentage cut-off of <1.13%, established by Hudson et al.^3^ in their paper from 2018. Among our 13 cases of CMML, six patients (46.2%) were noted to have an MO3 percentage <1.13%. Meanwhile, only 8 out of 22 non-CMML patients (36.4%) had MO3 percentage <1.13%. There was no association between MO3 percentage and the diagnosis of CMML (*p* = 0.724). Hence, our study confirms the limitations of peripheral blood MFC in the diagnosis of CMML as highlighted by Pophali et al. Results from other publications assessing this issue are summarised in Supplementary Table 1 (refs. ^1–4^).

Using the MO1 and MO3 percentage cut-offs previously established, we were unable to reliably diagnose CMML. In fact, in our study there was a distinct subset of four CMML patients with MO1 percentage values significantly lower than the cut-off of 94%. Notably, among this were two patients with CMML-2 (with MO1 percentages of 35 and 74%) and one patient with CMML which transformed to secondary AML (with MO1 percentage of 58%). Similar subsets of CMML patients with low MO1 percentages were also reported by Hudson et al. and Selimoglu-Buet et al. Furthermore, Picot et al. also alluded to the changes observed in MO1, MO2 and MO3 distribution depending on when the testing was performed (24 vs 48 h after harvesting). Picot et al. commented that when the analysis was performed at 48 h after collection, even in patients with CMML, the monocyte subset profile resembled that seen in reactive monocytosis. However, among our six patients with CMML noted to have an MO1 percentage ≤94%, the majority of samples, i.e. five, were analysed within 24 h and one sample was processed between 24 and 48 h from collection. Therefore, in our real-world data, time to analysis does not appear to have been a significant factor that influenced results.

In summary, there is a clinical need to develop accurate, reliable, and cost-effective methods to distinguish monocytosis due to CMML from other reactive entities. Although monocyte subset repartitioning has shown promise in previous works, our study highlights some of its limitations in a real-world setting. In agreement with Pophali et al., we suggest that more research with larger sample sizes is required before monocyte subset analysis can be confidently applied to discriminate reactive monocytosis from CMML.

## Supplementary information

Supplemental Materials
