# Supplementary material for: The use of monocyte subset repartitioning by flow cytometry for diagnosis of chronic myelomonocytic leukaemia
Source: Blood Cancer J. 2021 Jan 7;11(1):6. doi: 10.1038/s41408-020-00401-3 (PMC7791046; doi:10.1038/s41408-020-00401-3)
Supplement: Supplementary file 1 — Supplemental Materials [file 41408_2020_401_MOESM1_ESM.docx]

**Supplementary Materials**

**Method of multi-parametric flow cytometry (MFC) for monocyte subset repartitioning**

Peripheral blood samples were subjected to MFC from < 24 hours to up to 160 hours after collection. The following methodology was used. Whole blood (0.5-1mL) was collected in EDTA tubes and subsequently, lysed using 14mLs of ammonium chloride for 5 minutes at 37°C. This solution was then spun at 400g for 5 minutes, the supernatant was removed, and the remaining sample was washed in 10mL of PBSA. The resulting cell pellet was then resuspended to a working concentration of 2 x 10^7^ cells/ml. Antibody staining was performed by adding 5uL of specific antibodies to 25uL of the 2 x 10^7^ cells/mL cell suspension in each tube, spinning and incubating tubes for 10 minutes at room temperature in the dark. Data was acquired using the BD FACS Canto II flow cytometer. Monocyte subsets were identified using Kaluza software (Beckman Coulter, USA). A CD45/ side scatter gate was set to locate the monocyte population and specific antibody combinations were used to identify and exclude other lineages; these were – CD24 to exclude granulocytes and B cells, CD16 to exclude neutrophils, CD2 to exclude T cells and CD56 to exclude NK cells. Based on the CD14 and CD16 expression, the monocytes were then divided into: MO1 (CD14+/CD16-), MO2 (CD14low/CD16+) and MO3 (CD14-/CD16+). Results are reported descriptively and Fishers Exact Test (IBM® SPSS® Statistics Version 26) was used to establish if there was any correlation between the percentage of classical monocytes and the diagnosis of CMML.

**Supplementary Tables/Figures:**

|  |  |  |  |  | **MO1 > 94%^2^** | | **MO3 < 1.13%** | |
| --- | --- | --- | --- | --- | --- | --- | --- | --- |
| **Author(s)** | **Year** | **Country** | **Sample Size (n)** | **CMML Cases (n)** | **Sn (%)** | **Sp (%)** | **Sn (%)** | **Sp (%)** |
| Selimoglu-Buet et al. | 2015 | France | 307^1^ | 86^1^ | 91.9^1^ | 94.1^1^ | NA | NA |
| Picot et al. | 2018 | France | 55 | 5 | 100 | 97 | NA | NA |
| Hudson et al. | 2018 | USA | 68 | 16 | 88 | 94 | 100 | 96 |
| Pophali et al. | 2019 | USA | 184 | 43 | 75 | 95.4 | 75 | 82.7 |
| Murali et al. | 2020 | Australia | 35 | 13 | 53.8 | 81.8 | 46.2 | 63.6 |

**Table 1:** Summary of recent studies looking at the diagnostic accuracy of peripheral blood MFC in the diagnosis of CMML. Sensitivity (Sn) and specificity (Sp) reported for MO1 percentage cut off > 94% and MO3 percentage cut off < 1.13%. NA: not available.

^1^ data from the validation cohort

^2,^ Picot et al. used an MO1 cut off > 95%
